# Expectation-Maximization Algorithm for the Calibration of Complex Simulator Using a Gaussian Process Emulator

**DOI:** 10.3390/e23010053

**Published:** 2020-12-31

**Authors:** Yun Am Seo, Jeong-Soo Park

**Affiliations:** 1 AI Weather Forecast Research Team, National Institute of Meteorological Sciences, Seogwipo 697-010, Korea; seoya98@korea.kr; 2 Department of Statistics, Chonnam National University, Gwangju 61186, Korea

**Keywords:** best linear unbiased predictor, code tuning, iterative algorithm, Latin-hypercube design, mean squared error, metamodel, numerical optimization, surrogate

## Abstract

The approximated non-linear least squares (ALS) tunes or calibrates the computer model by minimizing the squared error between the computer output and real observations by using an emulator such as a Gaussian process (GP) model. A potential defect of the ALS method is that the emulator is constructed once and it is no longer re-built. An iterative method is proposed in this study to address this difficulty. In the proposed method, the tuning parameters of the simulation model are calculated by the conditional expectation (E-step), whereas the GP parameters are updated by the maximum likelihood estimation (M-step). These EM-steps are alternately repeated until convergence by using both computer and experimental data. For comparative purposes, another iterative method (the max-min algorithm) and a likelihood-based method are considered. Five toy models are tested for a comparative analysis of these methods. According to the toy model study, both the variance and bias of the estimates obtained from the proposed EM algorithm are smaller than those from the existing calibration methods. Finally, the application to a nuclear fusion simulator is demonstrated.

## 1. Introduction

Modern researchers have attempted to develop and use simulation code instead of excessively expensive or infeasible physical experiments in many fields. Advanced computer technology has made it possible to realize very complicated simulations. In most cases, the simulation code contains several unknown parameters (or universal constants). A classic method for determining the universal constants in computer models is non-linear least squares estimation (NLSE). It makes the sum of the squared error between the real observations and the computer responses as minimum. The NLSE, however, will become too computationally expensive or infeasible in terms of time when the computer model is time-consuming to run. In such cases, a statistical emulator can be used to determine the universal constants in the computer model so that the simulator or emulator can represent the real experiments effectively well. This process is known as “code tuning” or “calibration” [1,2,3,4].

Code tuning is defined as the procedure of enhancing the consistency between code responses and experimental data via determining the parameters inside the simulation code [5]. A differentiation can be made between tuning parameters and calibration parameters [3]. However, we use these two terms (calibration and code tuning) interchangeably.

Our work focuses on calibration in a frequentist manner [6,7,8], rather than a Bayesian approach [2,9,10]. A Bayesian calibration with a bias correction was introduced by Kennedy and O’Hagan [2]. Contributions to this topic include the related works of sequential tuning [11], multivariate computer outputs [12], identifiability [13,14], multi-fidelity [9], good designs for calibration [10], GP models for big data [7], and various metamodeling applications [8,15,16].

Calibration has often been performed within a framework, in the literature, in which the code suffers from a systematic bias or discrepancy for any parameter values. This shows a view that the mathematical equations inside the simulator may not perfectly describe the real world [2,12]. This framework may be more realistic, but it is beyond the range of this study. Thus, presentation in this study is focused on a statistical method that does not include the simulation discrepancy [1]. It would be, however, possible to extend our framework when the shape of the bias were available.

Cox et al. [1] studied an approximated NLSE in the form of approximated nonlinear least squares (ALS) for calibration. They utilized the Gaussian process (GP) as an emulator (or a surrogate or metamodel) of the complex simulator. The ALS first builds the GP model from the computer data. Then, it regards the built GP model as if it were true simulator. This surrogation makes the NLSE computationally feasible. We adopt the GP model as an emulator of the complex simulator, in this study. The GP model has been used successfully in constructing a regression model for complex data [17,18,19,20,21] and in analyzing computer experiments [22,23,24,25].

A drawback of the ALS is that the emulator (a GP model) is constructed once from the computer data only and it is not re-modeled after that. To address this defect, Seo et al. [4] considered an iterative modification of the ALS, which they referred it as the “max-min” algorithm. The max-min algorithm improves the calibration accuracy and acquires stable results. In this study, we propose another iterative method based on the expectation-maximization (EM) algorithm. We anticipate that this new method is more effective than existing approaches, including the ALS, max-min algorithm, and likelihood-based calibration. The proposed method is compared with existing calibration methods using five toy-models, in which the true values are known a priori.

This report is arranged as follows: a GP model for simulation experiments is described in Section 2. Existing methods including the ALS, max-min algorithm, and likelihood-based calibration are briefly introduced in Section 3. EM-based calibration is presented in Section 4. In Section 5, a toy model study is described. The application to a nuclear fusion simulator is presented in Section 6. Discussions are outlined in Section 7, followed by a summary in Section 8. Further details including technical specifications are provided in the Appendix. Certain contents of this study are inevitably similar to those of Reference [4,6] because the problem setting of these studies was very similar.

## 2. GP Model for Computer Experiments

Sacks et al. [22] proposed the adoption of the GP model for the analysis of deterministic computer experiments. The GP model can be expressed as follows, for the response Y(t) at site t:

(1) $$Y\left(t\right)=\sum_{i=1}^{p}\beta_{i}f_{i}\left(t\right)+Z\left(t\right),$$

where $\beta_{i}$*s* are unknown regression coefficients, $\left\{f_{i}\left(\cdot \right)\right\}$ is a set of known functions, and Z(·) is the GP (random variables) with mean zero and covariances $\sigma_{Z}^{2}R\left(t\right)$. In the above equation, the first term indicates a regression function and the second part (Z(t)) stands for the departure from the assumed regression function. The stochastic process term allows the predictions (5) to interpolate the deterministic responses. For $t_{i}=\left\{t_{i1},...t_{id}\right\}$ and $t_{j}=\left\{t_{j1},...t_{jd}\right\}$, the covariance between $Z(t_{i})$ and $Z(t_{j})$ is denoted by

(2) $$\mathrm{Cov}\left(Z\left(t_{i}\right),Z\left(t_{j}\right)\right)=\;V\left(t_{i},t_{j}\right)=\;\sigma_{Z}^{2}R\left(t_{i},t_{j}\right),$$

where $\sigma_{Z}^{2}$ is the variance of Z(·) and $R(t_{i},t_{j})$ is a correlation function. We select the Gaussian correlation family [26]:

(3) $$R\left(t_{i},t_{j}\right)=\exp\left(-\sum_{k=1}^{d}\theta_{k}{\left(t_{ik}-t_{jk}\right)}^{2}\right),$$

where $\theta_{k}^{\prime }$*s* are non-negative parameters. We define $v^{\prime }\left(t_{0}\right)$ and $f^{\prime }\left(t_{0}\right)$ as

(4) $$v^{\prime }\left(t_{0}\right)=\left[V\left(t_{0},t_{1}\right),\ldots ,V\left(t_{0},t_{n}\right)\right],\;\;\;f^{\prime }\left(t_{0}\right)=\left[f_{1}\left(t_{0}\right),\ldots ,f_{p}\left(t_{0}\right)\right].$$

In this case, $v^{\prime }\left(t_{0}\right)$ is a correlation vector between the observed (or training) sites and a prediction site $t_{0}$, and $f^{\prime }\left(t_{0}\right)$ is a design (or functional) vector of $t_{0}$.

When the correlation function R(·,·) is assigned and the observations *y* at sites t are given, the best linear unbiased predictor (BLUP) of $Y(t_{0})$ is [26]

(5) $$\hat{Y}\left(t_{0}\right)=\left[v^{\prime }\left(t_{0}\right),f^{\prime }\left(t_{0}\right)\right]{\left[\begin{array}{cc} V & F\\ F^{\prime } & 0 \end{array}\right]}^{-1}\left[\begin{array}{c} y\\ 0 \end{array}\right]=f^{\prime }\left(t_{0}\right)\hat{\beta }+v^{\prime }\left(t_{0}\right)V^{-1}\left(y-F\hat{\beta }\right),$$

where V=V(t,t) is an n×n covariance metrix, $F={\left[f_{j}\left(t_{i}\right)\right]}_{1\leq i\leq n,1\leq j\leq p}$ is an n×p design matrix, and $\hat{\beta }={\left(F^{\prime }V^{-1}F\right)}^{-1}F^{\prime }V^{-1}y$ is the GLSE (generalized least squares estimator) of β. Furthermore, the hyper-parameters in *V* are generally unknown, and hence, we usually estimate the hyper-parameters using the MLE (maximum likelihood estimation) from the observations *y*. These estimates are subsequently plugged into (5), causing (5) to become the so-called empirical BLUP (EBLUP) of $Y(t_{0})$ [26]. We used the “DiceKriging” [27] package of the R program. A brief description of the MLE calculation is provided in Appendix A.

We can build the prediction models differently according to the combination of $\theta^{\prime }s$ and $\beta^{\prime }s$ in (1) and (3). We construct the following two models, in this study:

(6) $$\begin{array}{cc} & Y\left(x\right)=\beta_{0}+\beta_{1}t_{1}+\ldots +\beta_{d}t_{d}+Z\left(t\right)+\varepsilon ,\\ & Model\;1:with\;common\;\theta ,\;\;\;Model\;2:with\;d\;different\;\theta^{\prime }s. \end{array}$$

In the above, “common θ” indicates that *d* number of θ${}^{\prime }s$ are constrained to be a common $\theta_{c}$ so that $\theta_{1}=\theta_{2}=\cdots =\theta_{d}:=\theta_{c}$. The final error term (ϵ) is for the randomness or measurement error in the real experiments. The error term is not applied to the computer responses because only a deterministic computer model is considered in this study. Other models such as the one from variable selection algorithms [28,29,30] are of course usable. We recommend References [26,31] for further details on the GP model.

## 3. Existing Calibration Methods

### 3.1. Data Structure and Notations

The experimental data and computer data are subscrived by “E” and “C”, respectively, and the combined (both) data are denoted by “B”. Let τ be a vector of calibration parameters with the dimension *q* and $\underline{T}$ represent the input variables of the simulator corresponding to τ. We denote *X* for the experimental input variables with the dimension *p*. In addition, let $n_{C},\;n_{E}$ be the sample sizes and $n_{B}=n_{C}+n_{E}$. More details are presented in Appendix B.

### 3.2. ALS Method

In this subsection, we briefly describe the ALS method considered in Reference [1]. If the simulation code is computationally costly to run, it is very difficult to optimize numerically a certain quantity from the code directly in terms of time. In this case, the ALS employs the GP model as a surrogate or an emulator of the computer code. The ALS first estimates the GP parameters using the MLE for the simulation data. Thereafter, it regards the built GP model as if it were true simulator. The ALS determines $\hat{\tau }$ by minimizing the following approximated residual sum of squares (ARSS):

(7) $$ARSS\left(\tau \right)=\sum_{i=1}^{n_{E}}{\left[y_{E,i}-\hat{Y}\left(\tau ,x_{E,i}\right)\right]}^{2},$$

where $\hat{Y}\left(\tau ,x_{E}\right)$ is the EBLUP of $Y(x_{0})$, as in (5).

The advantage of the ALS method is that it does not need additional runs of the simulator to calculate ARSS(τ) after a GP surrogate has been constructed from a computer dataset. Because there is no explicit minimizer in ARSS(τ), we employ the quasi-Newton iteration in the “optim” package of the R program.

A potential disadvantage of the ALS method is that the emulator (a GP model) is constructed once from the simulation data and it is no longer re-built. To address this defect, an iterative method of the ALS, namely the max-min algorithm, was considered by Seo et al. Reference [4].

### 3.3. Max-Min Algorithm

The tuning constants and GP parameters are alternately estimated by ALS method and the MLE, in the max-min algorithm. This method utilizes both experimental and computer data repeatedly until convergence. The steps are outlined as follows:

**Step 1**: Find the MLE of the GP parameters ${\hat{\theta }}^{\prime }s$, ${\hat{\beta }}^{\prime }s$, and ${\hat{\sigma }}_{Z}^{2}$ in Equations (2) and (6) using the simulation data ($T_{S}=\left(\tau_{S},x_{S}\right)$ and $y_{S}$) only.
**Step 2**: Determine $\hat{\tau }$, which minimizes the ARSS(τ) in (7) with the estimates ${\hat{\theta }}^{\prime }s$, ${\hat{\beta }}^{\prime }s$, and ${\hat{\sigma }}_{Z}^{2}$ from Step 1.
**Step 3**: [maximization] Find the MLE of the GP parameters ${\hat{\theta }}^{\prime }s$, ${\hat{\beta }}^{\prime }s$, and ${\hat{\sigma }}_{Z}^{2}$ using the combined data ($T_{B}$ and $y_{B}$), where $T_{B}=\left(\begin{array}{c} T_{E}^{*}\\ T_{S} \end{array}\right),y_{B}=\left(\begin{array}{c} y_{E}\\ y_{S} \end{array}\right),T_{E}^{*}={\left\{t_{E,1}^{*},...,t_{E,n_{E}}^{*}\right\}}^{\prime }$, and ${t_{E,i}^{*}}^{\prime }=\left(\hat{\tau },x_{E,i}\right)$.
**Step 4**: [minimization] Determine $\hat{\tau }$, which minimizes the ARSS(τ) in (7) with the estimates ${\hat{\theta }}^{\prime }s$, ${\hat{\beta }}^{\prime }s$, and ${\hat{\sigma }}_{Z}^{2}$ from Step 3.
**Step 5**: Repeat Step 3 and Step 4 until convergence, such as $\;\sum_{i=1}^{q}|{\hat{\tau }}_{i}^{old}-{\hat{\tau }}_{i}^{new}\left|\;/\;\right|{\hat{\tau }}_{i}^{old}|\;<\epsilon$.

In Steps 1 and 3, a GP model is constructed for the prediction. Steps 2 and 4 are the same in minimizing ARSS(τ), but Step 2 utilizes computer data only in the prediction $\hat{Y}\left(x_{E}\right)$, whereas Step 4 uses both data. In Step 3, $\hat{\tau }$ is the estimate obtained from the previous step. Steps 1 and 3 are the same in terms of seaching the MLE, but Step 1 utilizes computer data only, whereas Step 3 utilizes both data. Quasi-Newton numerical algorithms are employed for the optimizations in Steps 2–4.

Seo et al. [4] demonstrated the max-min method works better than the ALS. One defect of this method is that it needs more computing time than the ALS. Further details on this algorithm, including the stopping rule, can be found in [4].

### 3.4. Likelihood-Based Calibration

Cox et al. [1] considered the full likelihood function using the combined data for all parameters, including the calibration parameters τ; variance parameter $\sigma_{\epsilon E}^{2}$ and GP parameters θ, $\underline{\beta }$, and $\sigma^{2}$. Thus, all parameters are estimated simultaneously by the MLE method. This method is called as the full MLE, and it is applied to a GP model. The minus two times profile log likelihood function of all parameters is, without constants,

(8) $$-2\;log\;L\left(\tau ,\psi_{B};\;{\underline{y}}_{B},X_{B}\right)\;=\;n_{B}\;log\;\hat{\sigma_{B}^{2}}\;+\;log\;\left|V_{B}\right|,$$

where

(9) $$\hat{\sigma_{B}^{2}}={\left({\underline{y}}_{B}-F_{B}{\underline{\hat{\beta }}}_{B}\right)}^{t}V_{B}^{-1}\left({\underline{y}}_{B}-F_{B}{\underline{\hat{\beta }}}_{B}\right)/n_{B},$$

(10) $${\underline{\hat{\beta }}}_{B}\;=\;{\left({F_{B}}^{t}V_{B}^{-1}F_{B}\right)}^{-1}{F_{B}}^{t}V_{B}^{-1}{\underline{y}}_{B},$$

in which $\psi_{B}=\left({\underline{\theta }}_{B},{\underline{\beta }}_{B},\sigma_{B}^{2},\gamma_{E}\right)$ with ${\underline{\hat{\beta }}}_{B}$ and $\hat{\sigma_{B}^{2}}$ inserted, and $\gamma_{E}=\sigma_{\epsilon E}^{2}/\sigma^{2}$.

Cox et al. [1] considered other likelihood-based approaches. One method is the so-called separated MLE (SMLE). This maximizes the conditional likelihood of the experimental data given the simulation data. In this case, the GP parameters $\underline{\theta }$, $\underline{\beta }$, and $\sigma^{2}$ are determined by the marginal MLE from the computer data. These estimates are then inserted into in obtaining the conditional MLE. This result is subsequently maximized with respect to τ and $\gamma_{E}$, to acquire estimates of the above parameters. Advantages of likelihood-based methods for calibration are that they simultaneously or conditionally utilize the combined data, and so enrich the calibration methods. The SMLE was demonstrated to be superior to the full MLE in Reference [1]. Thus, in this study, we compare the SMLE with the proposed method. Further details of the SMLE are presented in Appendix C.

## 4. Proposed Method: EM-Based Calibration

An EM algorithm is an iterative method for determining the MLE or maximum a posteriori (MAP) estimates of parameters in statistical models [32], where the model usually depends on the unobserved latent variables. One example of the unobserved latent variables is the calibration parameters in computer experiments. The EM iteration alternates between an expectation (E) step and a maximization (M) step. The E-step calculates the expected value of the log likelihood at the current parameter estimates provided from the M-step. The M-step computes the parameters by maximizing the expected log likelihood determined in the E-step. These parameter estimates are subsequently employed to determine the distribution of the latent variables (or calibration parameters) in the following E-step [32].

The EM algorithm was described and given its name in 1977 in a paper by Dempster et al. [33]. Since then, the EM algorithm has been applied to many research areas, including computational statistics, machine learning, computer vision, hidden Markov models, item response theory, and computed tomography. See References [34,35,36] for further information on the EM algorithm and its applications.

To date, the EM algorithm has not been applied to the calibration problem of complex computer code. Because the tuning parameters in real experiments can be treated as unobserved latent variables, an EM algorithm may be appropriate to obtain the distribution of the tuning parameters. The steps of the proposed method for a given GP model are presented as follows:

**Initialization**: Provide initial values ($\hat{\tau }$) for τ from prior information on τ.
**M-step**: Determine the MLE ($\hat{\psi }$) of the GP parameters from the combined data in which $\hat{\tau }$ are inserted into the experimental data.
**E-step**: Set $\hat{\tau }$ as the conditional expectation of τ given the estimates ($\hat{\psi }$ from the M-step) of the GP parameters obtained under the combined data.
**Iterate**: E- and M-steps until convergence.

In the *k*-th iteration of the E-step, the conditional expectation of τ is actually the expectation of the posterior of τ:

(11) $$\begin{array}{cc} E(\tau^{*(k)}|y;x,\Psi^{\left(k\right)})\; & =\int \tau \;f(\tau |y;x,\Psi^{\left(k\right)})\;d\tau \\ \; & =\int \tau \;\frac{f(\tau ,y;x,\Psi^{\left(k\right)})}{f(y;x,\Psi^{\left(k\right)})}\;d\tau \\ \; & =\int \tau \;\frac{f\left(y|\tau ;x,\Psi^{\left(k\right)}\right)p\left(\tau ;x,\Psi^{\left(k\right)}\right)}{\int f\left(\tau ;x,\Psi^{\left(k\right)}\right)p\left(\tau ;x,\Psi^{\left(k\right)}\right)\;d\tau }d\tau \\ \; & =\int \tau \;\frac{G\times p(\tau ;x,\Psi^{\left(k\right)})}{\int G\times p(\tau ;x,\Psi^{\left(k\right)})d\tau }\;d\tau \\ \; & ⟹\;\tau^{*(k+1)}, \end{array}$$

where $G=f\left({\underline{y}}_{B}|\tau ;{\underline{x}}_{B},{\hat{\psi }}^{\left(k\right)}\right)=pdf\;(probability\;density\;function)\;of\;MN\left(F_{B}{\hat{\beta }}^{\left(k\right)},\;{\hat{\sigma }}^{2(k)}{V_{B}}^{\left(k\right)}\right)$, $\;\psi^{\left(k\right)}=\left({\underline{\theta }}^{\left(k\right)},{\underline{\beta }}^{\left(k\right)},\sigma^{2(k)}\gamma_{E}^{2(k)}\right)$, and $\;p(\tau ;{\underline{x}}_{B},{\hat{\psi }}^{\left(k\right)})$ is a prior (pdf) of τ. Note that $F_{B}$ and $V_{B}$ are functions of τ. A numerical integration method by Reference [37] in the R package “cubature” [38] was used for the calculation of (11). We set a uniform distribution as the prior of τ. Other priors can easily be incorporated.

A slightly modified likelihood function from (8) is employed to calculate the MLE in the M-step: $-2\;log\;L(\psi_{B};\;{\underline{y}}_{B},X_{B},\hat{\tau })$, where $\hat{\tau }$ is obtained from the E-step. The M-step is basically the same as Step 3 in the max-min algorithm. The major difference between the two algorithms is that the max-min minimizes the ARSS, whereas the EM calculates the conditional expectation. One defect of the EM method is that it requires more computing time than the ALS or Kennedy–O’Hagan (KOH) method [2].

In each iteration of the E- and M-steps, parameters in the emulator and the combined data are updated. We expect this updating procedure affects positively to the estimation of the calibration parameter. The EM and max-min use the combined data for constructing an emulator, whereas the ALS uses the computer data only. The addition of relevant data generally enhances the prediction capacity of the emulator.

It is worth noting that the EM algorithm converges effectively, according to our experience when executing it in the test function study. The median iteration number is approximately 10 (the first and third quartiles are 6 and 28 iterations, respectively) based on 10 trials for test function 1, as described in detail in the following section.

Table 1 presents a classification of the calibration methods based on the τ estimation, emulator building, and outer iteration. It can be observed that the EM algorithm can be viewed as an extension of the KOH method.

## 5. Test Function Study

In this section, we describe the application of the calibration methods to test functions (or toy models) in which the true tuning parameters were known a priori. A set of five toy models in different situations were arranged for a comparison of the methods. These test functions are simple toy models, i.e., easy to compute. However, we treated these functions as if they were time-consuming simulators.

The computer data and experimental data with sample sizes $n_{C}$ and $n_{E}$, respectively, were generated by

$$y_{C}=Y\left(\tau_{C},x_{C}\right),\;\;\;\;y_{E}=Y\left(\tau^{*},x_{E}\right)+e.$$

The five test functions along with $n_{E}=n_{C}=30$ and with the true constants of τ are described as follows:

$$\begin{array}{cc} \mathrm{Test}\mathrm{function}1:\;Y(\tau ,x) & =\tau_{1}\exp\left(\tau_{2}+x_{1}\right)+\tau_{1}x_{2}^{2}-\tau_{2}x_{3}^{2}\\ \mathrm{Computer}\;\mathrm{data}:\; & T_{1}∼U\left(0,5\right),\;T_{2}∼U\left(0,4\right),\\ \; & x_{1}∼U\left(-3,3\right),\;x_{2}∼U\left(-3,3\right),\;x_{3}∼U\left(0,6\right)\\ \mathrm{Experimental}\;\mathrm{data}:\; & \tau_{1}=2,\;\tau_{2}=2,\;\sigma_{E}^{2}=1. \end{array}$$

$$\begin{array}{cc} \mathrm{Test}\mathrm{function}2:\;Y(\tau ,x) & =\tau_{1}\exp\left(\tau_{2}+x_{1}+\tau_{3}\right)+\tau_{1}\tau_{3}x_{2}^{2}-\tau_{2}x_{3}^{2}-\tau_{3}\log\left(x_{4}\right)\\ \mathrm{Computer}\;\mathrm{data}:\; & T_{1}∼U\left(0,5\right),\;T_{2}∼U\left(0,4\right),\;T_{3}∼U\left(1,5\right),\\ \; & x_{1}∼U\left(-3,4\right),\;x_{2}∼U\left(-3,3\right),\;x_{3}∼U\left(0,6\right),\;x_{4}∼U\left(1,5\right)\\ \mathrm{Experimental}\;\mathrm{data}:\; & \tau_{1}=2,\;\tau_{2}=1,\;\tau_{3}=3,\;\sigma_{E}^{2}=1. \end{array}$$

$$\begin{array}{cc} \mathrm{Test}\mathrm{function}3:\;Y(\tau ,x) & =\tau_{1}\exp(|x_{1}+x_{2}\left|\right)+\tau_{2}\left(x_{4}+1.2x_{5}+1\right)/2.5+\tau_{3}\left(\tau_{2}+2x_{3}+x_{4}\right)\\ \; & +\tau_{4}\left(x_{1}+x_{3}-\tau_{4}x_{5}-x_{6}\right)+2\cos\left(6\left(x_{2}+x_{3}+x_{3}\right)\right)\\ \mathrm{Computer}\;\mathrm{data}:\; & T_{1}∼U\left(0,5\right),\;T_{2}∼U\left(0,3\right),\;T_{3}∼U\left(0,7\right),\;T_{4}∼U\left(0,6\right),\\ \; & x_{1}∼U\left(-2,6\right),\;x_{2}∼U\left(-6,6\right),\;x_{3}∼U\left(-5,6\right),\;x_{4}∼U\left(-6,6\right)\\ \; & x_{5}∼U\left(-6,4\right),\;x_{6}∼U\left(-6,3\right)\\ \mathrm{Experimental}\;\mathrm{data}:\; & \tau_{1}=2,\;\tau_{2}=1,\;\tau_{3}=4,\;\tau_{4}=3,\;\sigma_{E}^{2}=6. \end{array}$$

$$\begin{array}{cc} \mathrm{Test}\mathrm{function}4:\;Y(\tau ,x) & =\tau_{1}x_{1}^{2}+\tau_{2}x_{2}+\tau_{3}cos\left(x_{3}\pi \right)+\tau_{4}sin\left(x_{4}\pi \right)\\ \mathrm{Computer}\;\mathrm{data}:\; & T_{1}∼U\left(0,5\right),\;T_{2}∼U\left(0,5\right),\;T_{3}∼U\left(0,7\right),\;T_{4}∼U\left(0,5\right)\\ \; & x_{1}∼U\left(0,3\right),\;x_{2}∼U\left(0,3\right),\;x_{3}∼U\left(0,2\right),\;x_{4}∼U\left(0,2\right)\\ \mathrm{Experimental}\;\mathrm{data}:\; & \tau_{1}=1,\;\tau_{2}=2,\;\tau_{3}=3,\;\tau_{4}=2,\;\sigma_{E}^{2}=4. \end{array}$$

$$\begin{array}{cc} \mathrm{Test}\mathrm{function}5:\;Y(\tau ,x) & =exp\left(\tau_{2}x_{1}+x_{2}\right)/\left(3+\tau_{1}\right)+\tau_{2}\sqrt{\left(x_{4}+x_{5}\right)}+\tau_{1}{\left(x_{3}+x_{6}\right)}^{2}\\ \mathrm{Computer}\;\mathrm{data}:\; & T_{1}∼U\left(0,5\right),\;T_{2}∼U\left(1,5\right)\\ \; & x_{1}∼U\left(-0.5,0.5\right),\;x_{2}∼U\left(-0.5,0.5\right),\;x_{3}∼U\left(-0.5,0.5\right),\\ \; & x_{4}∼U\left(0,2\right)\;x_{5}∼U\left(0,2\right),\;x_{6}∼U\left(0,1\right)\\ \mathrm{Experimental}\;\mathrm{data}:\; & \tau_{1}=2,\;\tau_{2}=2,\;\sigma_{E}^{2}=0.25. \end{array}$$

Optimal Latin hypercube designs [39,40] were used for sampling in the independent variables for real experiments ($x_{E}$) and for computer experiments $\left(\tau_{S},x_{S}\right)$. A total of 30 different designs for computer data were employed for each test function to take into account uncertainty in the design, whereas the real experimental design was fixed.

As a result, the average of estimates and the standard deviations from 30 trials are presented. The averaged Euclidean distance from the estimates to the true values was computed to evaluate the performance of the methods. (In addition to the Euclidean distance, one can consider an weighted distance such as the Mahalanobis distance [41]. It may be more meaningful than the Euclidean distance in the sense that the Mahalanobis distance takes into account the covariances among estimates of calibration parameters. The weighted distance is not considered in this study, but may be useful in the future study.) The root mean squared error (RMSE) of the estimates is also provided. The formula for the RMSE is as follows:

(12) $$RMSE\left(\hat{\tau }\right)=\sqrt{Bias^{2}\left(\hat{\tau }\right)+\sum_{i=1}^{q}\left(std({\hat{\tau }}_{i}\right){)}^{2}},$$

where $Bias(\hat{\tau })$ is the averaged Euclidean distance to the true constants and $std({\hat{\tau }}_{i})$ is the standard deviation of each estimate obtained from 30 replications.

**Figure 1:**
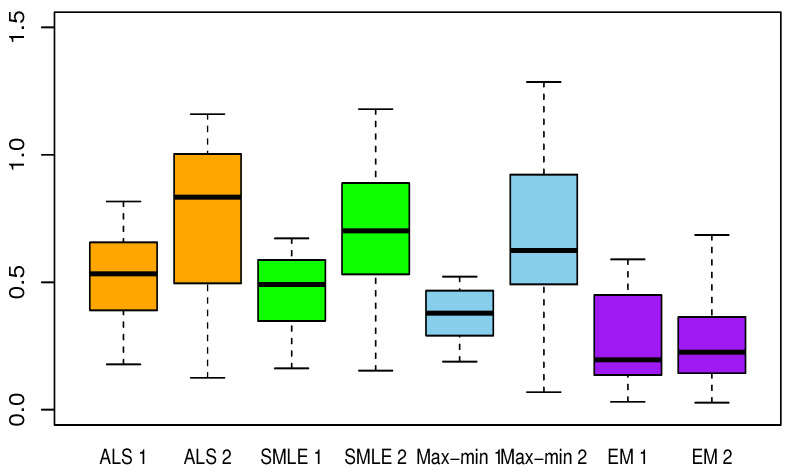
Boxplots of the Euclidean distances between true constants and estimated values from the calibration methods for test function 1, calculated from 30 random trials. The number 1 or 2 following each method stands for the Gaussian process Model 1 or Model 2. ALS: approximated nonlinear least squares estimation, SMLE: separated maximum likelihood estimation, EM: expectation and maximization algorithm, Max-min: max-min algorithm described in Section 3.3.

**Figure 2:**
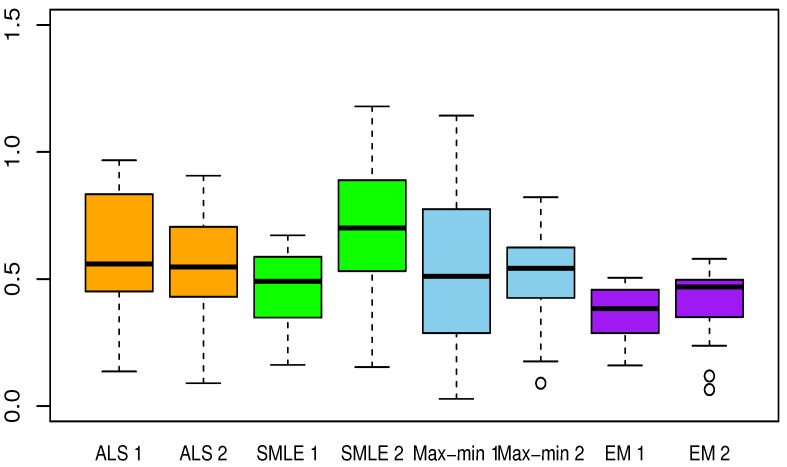
Same as Figure 1 but for test function 2.

**Figure 3:**
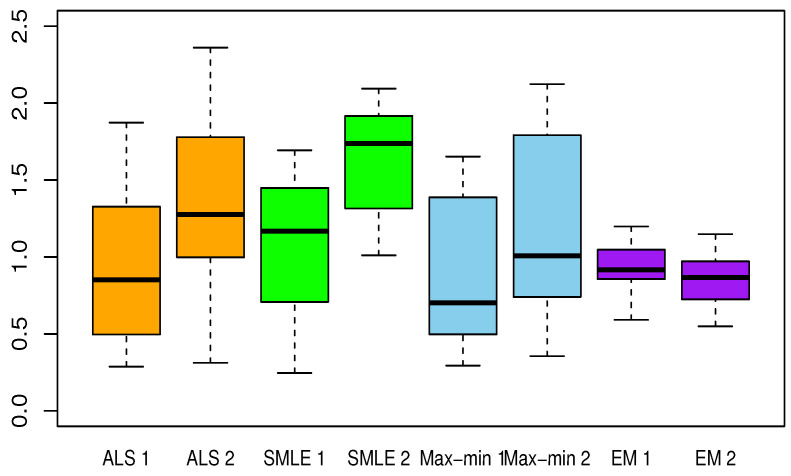
Same as Figure 1 but for test function 3.

**Figure 4:**
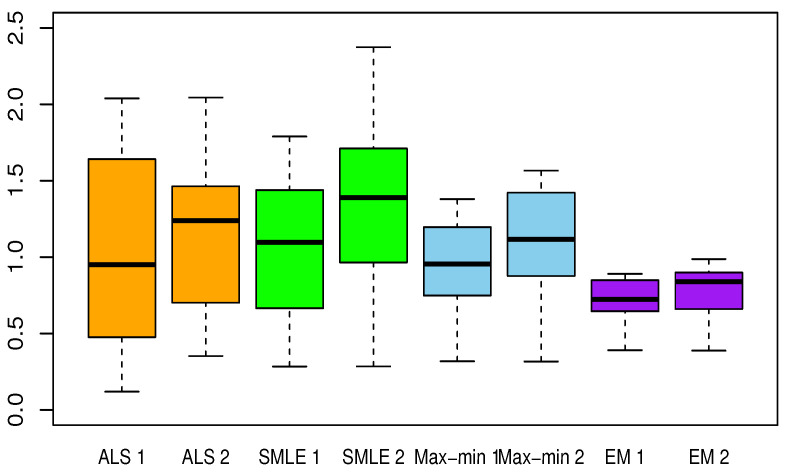
Same as Figure 1 but for test function 4.

**Figure 5:**
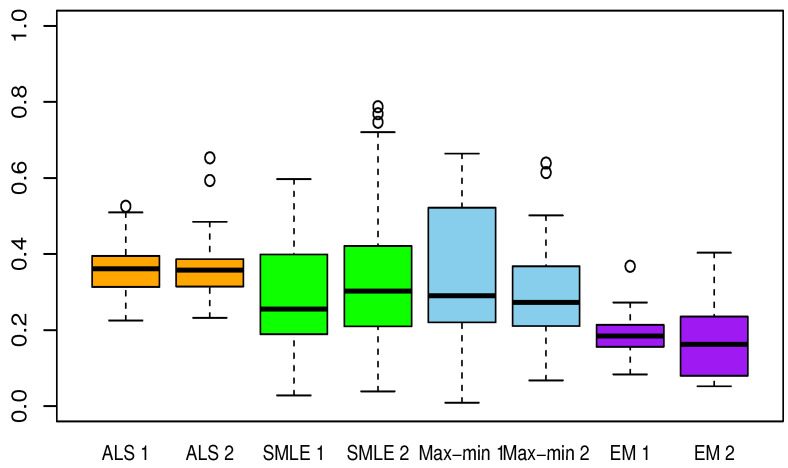
Same as Figure 1 but for test function 5.

Table 2, Table 3, Table 4, Table 5 and Table 6 present the results for each test function. The averaged Euclidean distance from the estimates to the true constants and the RMSEs of the estimates are displayed in each table. The standard deviations are the numbers in parentheses. In terms of the RMSE, the proposed method offered superior results over the ALS, SMLE, and max-min methods. In particular, the EM shows less bias and lower RMSE than the other methods. We have this result again in Figure 1, Figure 2, Figure 3, Figure 4 and Figure 5, which present the boxplots of the distance to the true value for each of the five test functions. In many cases, the medians of the EM estimates were nearer to the true constants than those from the SMLE, ALS, and max-min methods. The box lengths of the EM estimates were shorter than those obtained from the other methods. It is notable that the max-min was more effective than the ALS and SMLE in test functions 2–4. One plausible reason that the EM was superior to the max-min is that the numerical integration for the conditional expectation in the EM may be more stable than the numerical optimization of the ARSS in the max-min.

The computing times of the max-min and EM algorithms were much longer than those of the ALS and SMLE (see Table 7). The times were obtained using a personal computer with an Intel i5 CPU (3.6 GHz) and 16 Giga bytes of memory. Thus, we faced the limitation in extending our test functions to a higher-dimensional calibration parameters because of the heavy computing time in the max-min and EM algorithms. Numerical integration method for more than 10 dimensions may not be practical in the EM algorithm. In such high-dimensional cases, a Monte Carlo integration technique would be useful, but it requires more computing time.

## 6. Application to Nuclear Fusion Simulator

We present, in this section, the application of the calibration methods to computer code known as “Baldur” [42], which is a time-consuming simulator for the energy confinement time in a nuclear fusion device. It is called a “tokamak” in the Russian language. The mathematical model is simply expressed as follows, for a known complex function *f* calculated from the Baldur code:

(13) $$y=f(\tau_{1},\tau_{2},\tau_{3},\tau_{4},x_{1},x_{2},x_{3},x_{4}),$$

where $x_{1}$ is the input heating power, $x_{2}$ is the toroidal plasma current, $x_{3}$ is the line average electron density, and $x_{4}$ is the toroidal magnetic field. Calibration parameters $\tau =(\tau_{1},\tau_{2},\tau_{3},\tau_{4})$ determine the energy transfer, where each parameter is respectively, related to drift waves, rippling, resistive ballooning, and the critical value of $\eta_{i}$ [42].

The experimental data consisted of $x=\{x_{1},x_{2},x_{3},x_{4}\}$ with sample size 42 from the Poloidal Divertor Experiment (PDX) tokamak at Princeton. The computer data consisted of (τ,x) with sample size 64 from the Baldur code. The details on data can be found in References [1,42].

Table 8 presents the results of the τ estimation when using the ALS, SMLE, max-min, and EM algorithms on the basis of GP Model 1 and Model 2 from the tokamak data. The results were obtained using R program on a personal computer with an Intel i5 CPU (3.6 GHz) and 16 Giga bytes of memory.

**Figure 6:**
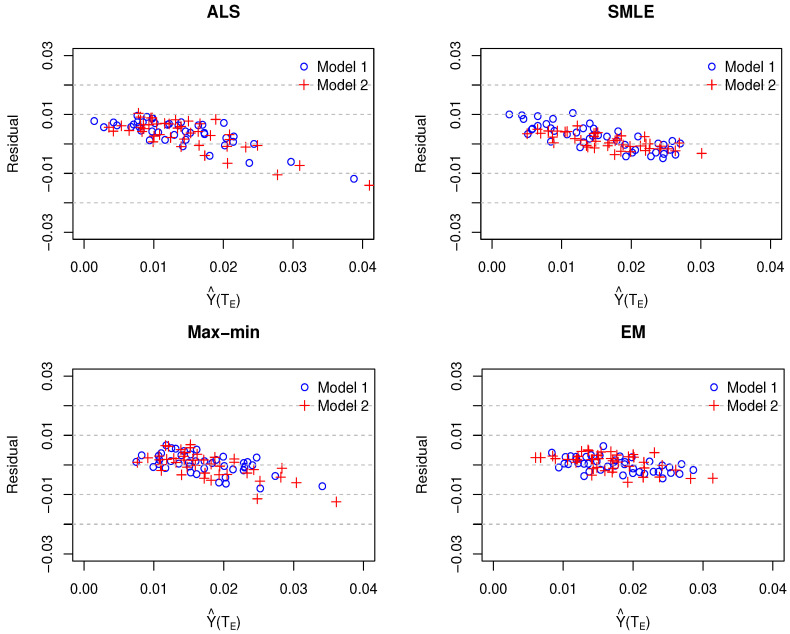
Residual plots for nuclear fusion data in which the simulation code was calibrated by various methods (ALS, SMLE, Max-min, and EM) using Gaussian process Model 1 or Model 2. The X-axes represents the predicted values $\hat{Y}\left(T_{E}\right)$ and the Y-axes represents the residuals ($y_{E}-\hat{Y}\left(T_{E}\right)$). The acronyms are same as Figure 1.

Figure 6 depicts the residuals ($y_{E}-\hat{Y}\left(T_{E}\right)$) plotted according to the predicted values $\hat{Y}\left(T_{E}\right)$ that were obtained by various methods using GP Model 1 and Model 2. The residual plots from all methods exhibited linear trends. The trend for the EM algorithm was the lowest among the methods.

## 7. Discussion

Certain basic limitations exist when calibrating computer models to real data. We have experienced that the performance of the calibration methods was influenced significantly by the designs of both the physical and computer experiments [43,44]. Thus, a sequential designing approach must be very useful in practice [10,11,16]. Relevant experimental designs under a sequential tuning will improve the calibration very well.

Iterative versions of calibration methods, including the KOH method, may be available. For example, an iterative version of the SMLE method can be summarized as follows:

Step 1: Acquire $\hat{\tau }$ by maximizing the conditional likelihood function of τ on the E-data, given $\left(y_{C},\;{\hat{\psi }}_{C}\right)$.
Step 2: [maximization] Acquire ${\hat{\psi }}_{B}$ by maximizing the likelihood from the both data, in which $\hat{\tau }$ are inserted into the E-data.
Step 3: [maximization] Acquire $\hat{\tau }$ by maximizing the conditional likelihood function of τ on the E-data, given $\left(y_{C},\;{\hat{\psi }}_{B}\right)$.
Step 4: Iterate Steps 2 and 3 until convergence.

The estimates $\hat{\tau }$ may vary according to the selected emulator. Thus an importance in calibration is the selection of the GP surrogate with some regression variables and GP parameters. We only used simple models (6) in this study. If the optimal GP model is built by the model selection algorithm, the result could be different.

## 8. Summary

The ALS method using a GP emulator is a basic calibration technique for complex computer models. However, it exhibits the potential drawback that the emulator is constructed once and it is no longer re-built. To overcome this defect, an iterative (EM) algorithm has been proposed in this study. The calibration parameters of the simulation code are calculated by the conditional expectation (E-step), whereas the GP parameters are updated by maximum likelihood estimation (M-step). These EM steps are alternately repeated until convergence by using both computer and experimental data.

We examined five test functions for a performance comparison. We confirmed that our proposed method (the EM algorithm) provided better results than the SMLE, ALS, and max-min methods in terms of the RMSE. The disadvantage of the proposed method is that it is more time-consuming than the ALS, because the EM algorithm needs to optimize complex functions and compute the conditional expectation using numerical integration based on the combined data. Nonetheless, the EM method can provide improved calibration as well as a superior emulator of the computer code compared to non-iterative methods, including the ALS and SMLE.

## Figures and Tables

**Table 1:** A classification of the calibration methods based on τ estimation, emulator building, and the number of outer iterations. Acronyms: NLSE = nonlinear least squares estimation, ALS = approximated NLSE, MLE (A) = maximum likelihood estimation calculated from data A, FMLE = full MLE, SMLE = separated MLE, Cond Expect (D) = conditional expectation based on data D, EM = expectation and maximization algorithm, Max-min = max-min algorithm described in Section 3.3, KOH = Kenney-O’Hagan method.

Method	τ Estimation	Emulator Building	Outer Iterations
NLSE	NLSE		1
ALS	ALS (E)	MLE (C)	1
FMLE	MLE (C, E, $\psi_{B}$ ) (all together)		1
SMLE	MLE (C|E)	MLE (C)	1
EM	Cond Expect (C, E)	MLE (C, E)	many
Max-min	ALS (E)	MLE (C, E)	many
KOH	Cond Expect (E)	MLE(C)	1

**Table 2:** Result for the test function 1 with true constants $\tau_{1}^{*}=2$, $\tau_{2}^{*}=2$. The values presented are the averaged estimates of $\tau^{*}$, the averaged Euclidean distance from the estimates to the true constants, the root mean squared error (RMSE) of the estimates. The standard deviations are in parentheses. The acronyms are same as Table 1. The number 1 or 2 following each method stands for the Gaussian process Model 1 or Model 2.

Method	Average of ${\hat{\tau }}_{1}$	Average of ${\hat{\tau }}_{2}$	Average Distance	RMSE
ALS 1	1.609 (0.207)	1.773 (0.246)	0.527 (0.168)	0.617
ALS 2	1.448 (0.249)	1.602 (0.351)	0.749 (0.288)	0.864
SMLE 1	1.790 (0.245)	1.758 (0.275)	0.462 (0.147)	0.591
SMLE 2	2.228 (0.409)	1.465 (0.267)	0.703 (0.280)	0.856
Max-min 1	2.296 (0.135)	1.966 (0.224)	0.377 (0.109)	0.459
Max-min 2	2.382 (0.399)	2.151 (0.575)	0.718 (0.342)	1.003
EM 1	2.024 (0.212)	1.828 (0.204)	**0.276** (0.197)	**0.103**
EM 2	2.098 (0.234)	2.894 (0.166)	**0.264** (0.177)	**0.390**

**Table 3:** Same as Table 2 but for test function 2 with true values $\tau_{1}^{*}=2$, $\tau_{2}^{*}=1$, $\tau_{3}^{*}=3$.

Method	Average of ${\hat{\tau }}_{1}$	Average of ${\hat{\tau }}_{2}$	Average of ${\hat{\tau }}_{3}$	Average Distance	RMSE
ALS 1	1.801 (0.324)	1.349 (0.225)	3.301 (0.319)	0.598 (0.232)	0.784
ALS 2	1.715 (0.244)	1.107 (0.322)	3.034 (0.344)	0.570 (0.209)	0.779
SMLE 1	1.737 (0.565)	1.080 (0.527)	2.957 (0.519)	0.873 (0.398)	1.276
SMLE 2	1.912 (0.347)	0.901 (0.381)	2.853 (0.416)	0.585 (0.353)	0.884
Max-min 1	1.706 (0.293)	0.842 (0.127)	3.003 (0.435)	0.535 (0.327)	0.760
Max-min 2	1.768 (0.231)	1.106 (0.314)	3.054 (0.283)	0.511 (0.176)	0.702
EM 1	2.108 (0.238)	1.207 (0.116)	2.975 (1.164)	**0.371** (0.109)	**0.484**
EM 2	2.163 (0.239)	1.261 (0.160)	2.980 (0.112)	**0.414**(0.128)	**0.516**

**Table 4:** Same as Table 2 but for test function 3 with true values $\tau_{1}^{*}=2$, $\tau_{2}^{*}=1$, $\tau_{3}^{*}=4$, $\tau_{4}^{*}=3$.

Method	Average of ${\hat{\tau }}_{1}$	Average of ${\hat{\tau }}_{2}$	Average of ${\hat{\tau }}_{3}$	Average of ${\hat{\tau }}_{4}$	Average Distance	RMSE
ALS 1	2.314 (0.408)	1.185 (0.335)	3.911 (0.578)	3.367 (0.571)	0.963 (0.514)	1.366
ALS 2	2.224 (0.587)	1.175 (0.341)	3.713 (0.865)	3.323 (0.793)	1.324 (0.547)	1.895
SMLE 1	1.923 (0.465)	1.316 (0.472)	3.713 (0.515)	3.309 (0.648)	1.080 (0.443)	1.513
SMLE 2	1.861 (0.446)	1.365 (0.643)	3.423 (0.749)	3.541 (0.655)	1.609 (0.347)	2.047
Max-min 1	2.060 (0.321)	1.166 (0.300)	3.626 (0.553)	3.315 (0.528)	**0.902**(0.463)	1.261
Max-min 2	2.039 (0.544)	1.469 (0.339)	3.753 (0.785)	2.845 (0.726)	1.208 (0.612)	1.736
EM 1	2.583 (0.198)	1.522 (0.119)	3.668 (0.250)	3.029 (0.254)	0.938 (0.139)	**1.030**
EM 2	2.480 (0.239)	1.403 (0.252)	3.681 (0.325)	3.024 (0.223)	**0.859** (0.158)	**1.007**

**Table 5:** Same as Table 2 but for test function 4 with true values $\tau_{1}^{*}=1$, $\tau_{2}^{*}=2$, $\tau_{3}^{*}=3$, $\tau_{4}^{*}=2$.

Method	Average of ${\hat{\tau }}_{1}$	Average of ${\hat{\tau }}_{2}$	Average of ${\hat{\tau }}_{3}$	Average of ${\hat{\tau }}_{4}$	Average Distance	RMSE
ALS 1	0.935 (0.237)	1.753 (0.396)	3.304 (0.908)	1.951 (0.610)	1.025 (0.628)	1.568
ALS 2	0.874 (0.250)	1.852 (0.414)	2.765 (0.846)	1.729 (0.672)	1.149 (0.456)	1.650
SMLE 1	0.845 (0.291)	1.975 (0.758)	2.875 (0.532)	1.852 (0.607)	1.069 (0.437)	1.566
SMLE 2	1.008 (0.515)	1.895 (0.895)	2.740 (0.475)	1.816 (0.953)	1.398 (0.53)	2.038
Max-min 1	0.562 (0.261)	2.155 (0.472)	3.087 (0.549)	2.049 (0.560)	0.955 (0.291)	1.348
Max-min 2	0.600 (0.230)	2.319 (0.549)	3.114 (0.749)	2.104 (0.407)	1.095 (0.362)	1.510
EM 1	1.160 (0.198)	2.017 (0.296)	3.383 (0.190)	2.418 (0.169)	**0.718** (0.134)	**0.841**
EM 2	0.921 (0.268)	1.863 (0.397)	3.379 (0.248)	2.498 (0.160)	**0.797** (0.149)	**0.976**

**Table 6:** Same as Table 2 but for test function 5 with true values $\tau_{1}^{*}=2$, $\tau_{2}^{*}=3$.

Method	Average of ${\hat{\tau }}_{1}$	Average of ${\hat{\tau }}_{2}$	Average Distance	RMSE
ALS 1	1.796 (0.274)	2.076 (0.130)	0.361 (0.078)	0.471
ALS 2	1.745 (0.253)	2.060 (0.105)	0.365 (0.093)	0.456
SMLE 1	2.025 (0.307)	2.038 (0.116)	0.287 (0.157)	0.436
SMLE 2	2.157 (0.483)	2.041 (0.154)	0.436 (0.295)	0.667
Max-min 1	2.074 (0.374)	2.001 (0.100)	0.340 (0.192)	0.515
Max-min 2	1.889 (0.292)	2.045 (0.125)	0.298 (0.152)	0.436
EM 1	2.054 (0.140)	2.085 (0.098)	**0.189** (0.054)	**0.255**
EM 2	2.030 (0.143)	2.102 (0.085)	**0.172**(0.094)	**0.239**

**Table 7:** Averaged computing time in seconds for tuning methods with $n_{E}=n_{C}=30$ from 30 trials for each of test functions 2, 4, and 5.

Function	No. of τ	No. of *X*	ALS	SMLE	Max-Min	EM
Test 2	3	4	149	150	661	471
Test 4	4	6	185	295	1207	1300
Test 5	2	6	23	117	435	320

**Table 8:** Estimates of the tuning parameters by various calibration methods from the nuclear fusion data. The last column shows computing time in seconds for tuning methods. The acronyms are same as Figure 1.

Method	${\hat{\tau }}_{1}$	${\hat{\tau }}_{2}$	${\hat{\tau }}_{3}$	${\hat{\tau }}_{4}$	Computing Time
ALS 1	1.012	2.035	1.110	1.308	337
ALS 2	1.195	1.593	0.956	1.035	1017
SMLE 1	1.120	2.055	0.118	1.303	585
SMLE 2	1.290	1.342	1.204	1.157	1230
Max-min 1	0.670	1.125	0.468	1.826	1674
Max-min 2	0.732	2.592	1.311	1.034	8042
EM 1	−0.459	3.227	1.750	1.407	1481
EM 2	1.064	2.428	1.048	1.030	7717

## Abbreviations

NLSE	non-linear least squares estimation
GP	Gaussian process
ALS	approximated NLSE
ARSS	approximated residual sum of squares
E-data	real experimental data
C-data	computer data
B-data	both of E-data and C-data
MLE	maximum likelihood estimation
FMLE	full MLE
SMLE	separated MLE
EM	expectation and maximization
E-step	expectation step
M-step	maximization step
pdf	probability density function
Max-min	maximization and minimization algorithm
KOH	Kenney-O’Hagan method
RMSE	root mean squared error

## Appendix A. Maximum Likelihood Estimation in GP Model

Once the data have been obtained at the design sites $\left\{{\underline{x}}_{1},...,{\underline{x}}_{n}\right\}$, we employ the MLE method to estimate the GP parameters. Because we assume that y(x) is a GP with mean $F\underline{\beta }$ and covariance $\sigma^{2}\;R$ for a design matrix *F*, the likelihood function of $\underline{y}$ is

(A1) $$L\left(\underline{y};\;\underline{\theta },\underline{\beta },\sigma^{2},\gamma_{C},\;\underline{x}\right)\;=\;\frac{{\left(2\pi \sigma^{2}\right)}^{-n/2}}{\sqrt{\left|\;V\right|}}\;exp\left(\;-\;\frac{{\left(\underline{y}-F\underline{\beta }\right)}^{t}V^{-1}\left(\underline{y}-F\underline{\beta }\right)}{2\;\sigma^{2}}\;\right).$$

When the hyper-parameters $\underline{\theta }$ and $\gamma_{C}$ are specified, the MLEs of $\sigma^{2}$ and $\underline{\beta }$ are

(A2) $$\underline{\hat{\beta }}\;=\;{\left(F^{t}V^{-1}F\right)}^{-1}F^{t}V^{-1}\underline{y},\;\;\;\;\;\;\hat{\sigma^{2}}\;=\;\frac{1}{n}{\left(\underline{y}-F\underline{\hat{\beta }}\right)}^{t}V^{-1}\left(\underline{y}-F\underline{\hat{\beta }}\right).$$

Because there is not a closed-form solution from the likelihood equations, we used a numerical optimization routine. The Cholesky decomposition $V=U^{t}U$ is used as a major computation in calculating the likelihood function, where *U* is an upper triangular Cholesky factor.

## Appendix B. Data Structure and Notations for Calibration

### Appendix B.1. Notations

The following notations are used for the computer and experimental data with $n_{C}$ and $n_{E}$ observations:

*T*: input variables T=(τ,x) of computer code with *d* dimensions
τ: *q*-dimensional calibration parameters
$\tau_{C}$: *q*-dimensional input variables of computer code corresponding to calibration parameters τ
$x_{C}$: *d*-*q* dimensional independent variables in computer code
Y(τ,x) or $y_{C}$: responses from simulator for input variables (τ,x)
$x_{E}$: independent variables in real experiments with *d*-*q* dimensions
$y_{E}$: observations in real experiments

The subscripts *C* and *E* indicate the computer and real experiments, respectively. If the simulator mimics the real experimental data effectively well without discrepancy, we use the following model to approximate $y_{E}$:

(A3) $$y_{E}=Y\left(\tau ,x_{E}\right)+e,$$

where *e* is independent and normally distributed random variable with mean zero and variance $\sigma_{E}^{2}$. Certain contents of this Appendix are similar to those in References [4,6].

### Appendix B.2. Computer and Experimental Data

We have the data matrix of the independent variables $X_{C}$ and $X_{E}$ for the computer and experimental data, respectively:

(A4) $$X_{E}\;=\;\left[\begin{array}{cccccccc} \tau_{1} & \tau_{2} & \cdots & \tau_{q} & x_{E11} & x_{E21} & \cdots & x_{Ep1}\\ \tau_{1} & \tau_{2} & \cdots & \tau_{q} & x_{E12} & x_{E22} & \cdots & x_{Ep2}\\ \vdots & & & \vdots & & & & \vdots \\ \tau_{1} & \tau_{2} & \cdots & \tau_{q} & x_{E1n_{E}} & x_{E2n_{E}} & \cdots & x_{Epn_{E}} \end{array}\right]$$

(A5) $$X_{C}\;=\;\left[\begin{array}{cccccccc} t_{11} & t_{21} & \cdots & t_{q1} & x_{C11} & x_{C21} & \cdots & x_{Cp1}\\ t_{12} & t_{22} & \cdots & t_{q2} & x_{C12} & x_{C22} & \cdots & x_{Cp2}\\ \vdots & & & \vdots & & & & \vdots \\ t_{1n_{C}} & t_{2n_{C}} & \cdots & t_{qn_{C}} & x_{C1n_{C}} & x_{C2n_{C}} & \cdots & x_{Cpn_{C}} \end{array}\right].$$

In the above, $t_{ij}$ in $X_{C}$ represents the *j*-th value of the *i*-th *T* variable ($T_{i}$), whereas $x_{Eij}$ and $x_{Cij}$ denote the *j*-th value of the *i*-th *X* variable of the experimental ($X_{Ei}$) and computer ($X_{Ci}$) input. Furthermore, $X_{E}$ is a $n_{E}\times \left(q+p\right)$ matrix and $X_{C}$ is a $n_{C}\times \left(q+p\right)$ matrix. Note that the first part of $X_{E}$ is composed of the unknown parameters $\tau_{1},\cdots ,\tau_{q}$, whereas the corresponding part of $X_{C}$ comprises the input values ($t_{ij}$).

### Appendix B.3. Combined Data

The following notations are introduced for the combined computer and experimental data:

(A6) XB=XCXE,FB=FCFE=f(XC)f(XE),y_B=y_Cy_E.

$X_{B}$ is the design matrix of the independent variables. $F_{B}$ is defined as the functions of the input variable values. ${\underline{y}}_{B}$ is a vector of the combined responses. Note that $\underline{T}$ are the input varaibles of the computer code. In this case, $X_{C}$ and $F_{C}$ contain $\underline{T}$, whereas $X_{E}$ and $F_{E}$ are the functions of the tuning parameters τ.

The GPM is subsequently applied to the computer and experimental data simultaneously. Let $\psi =(\tau ,\underline{\theta },\gamma_{C},\gamma_{E},\sigma^{2},\underline{\beta })$, where $\gamma_{C}=\sigma_{\epsilon C}^{2}/\sigma^{2}$ and $\gamma_{E}=\sigma_{\epsilon E}^{2}/\sigma^{2}$. Here, $\sigma_{\epsilon C}^{2}$ and $\sigma_{\epsilon E}^{2}$ are the variances of the error term (ϵ) in the GP model for the computer and experimental data, respectively. We set $\sigma_{\epsilon C}^{2}=0$, and thus, $\gamma_{C}=0$, because only a deterministic computer model is considered in this study. When necessary, ${\underline{\beta }}_{C}$ and ${\underline{\beta }}_{E}$ are used to denote the regression coefficients for the computer and real experimental data. Thus, given the independence and normality assumptions, we have

(A7) $$Law\;\left({\underline{y}}_{B}|\psi \right)=N\left(F_{B}{\underline{\beta }}_{B},\;V_{B}\right),$$

where

(A8) $${\underline{\beta }}_{B}={\left({\underline{\beta }}_{C},\;{\underline{\beta }}_{E}\right)}^{t},$$

(A9) $$V_{B}\;=\left[\begin{array}{cc} V_{CC} & V_{CE}\\ V_{EC} & V_{EE} \end{array}\right]=\;\sigma^{2}\left[\begin{array}{cc} R(X_{C},X_{C}) & R(X_{C},X_{E})\\ R(X_{E},X_{C}) & R(X_{E},X_{E}) \end{array}\right]\;+\;\sigma^{2}\left[\begin{array}{cc} \gamma_{C}I & 0\\ 0 & \gamma_{E}I \end{array}\right],$$

in which $R(X_{C},X_{E})$ represents a $n_{C}\times n_{E}$ correlation matrix computed between $X_{C}$ and $X_{E}$. $V_{B}$ is a $n_{B}\times n_{B}$ positive definite covariance matrix for the combined data, where $n_{B}=n_{C}+n_{E}$.

## Appendix C. Separated MLE for Calibration

For the details of the SMLE, we utilize the conditional distribution of the experimental data given the computer data, which is normally distributed with a mean

(A10) $$\mu_{E|C}\;=\;E\left[{\underline{y}}_{E}|{\underline{y}}_{C};\;\tau ,\psi \right]\;=\;F_{E}{\underline{\beta }}_{E}+\;+\;V_{CE}^{t}\;V_{CC}^{-1}\;\left({\underline{y}}_{C}-F_{C}{\underline{\beta }}_{C}\right)$$

and covariance

(A11) $$V_{E|C}\;=\;Cov\left[{\underline{y}}_{E}|{\underline{y}}_{C};\;\tau ,\psi \right]\;=\;V_{EE}\;-\;V_{CE}^{t}\;V_{CC}^{-1}\;V_{CE},$$

where the covariance matrices *V* are given as per (A9). In these formulae, we suppress the parameter dependencies in $\mu_{E}=F_{E}{\underline{\beta }}_{E}$, $\mu_{C}=F_{C}{\underline{\beta }}_{C}$, $V_{CE}$, $V_{CC}$, and $V_{EE}$. Thus, the minus two times concentrated (without constants) log conditional likelihood function with $\underline{\hat{\beta }}$ and ${{\hat{\sigma }}^{2}}_{E|C}$ plugged in is

(A12) $$-2\;log\;L\left(\tau ,\gamma_{E};X_{E},{\underline{y}}_{E}|\;{\underline{y}}_{C},{\hat{\gamma }}_{C},{\underline{\hat{\beta }}}_{C},\hat{\underline{\theta }},X_{C}\right)\;=\;n_{E}\;log\;{{\hat{\sigma }}^{2}}_{E|C}\;+\;log\;\left|V_{E|C}\right|,$$

where

(A13) $${{\hat{\sigma }}^{2}}_{E|C}={\left({\underline{y}}_{E}-{\hat{\mu }}_{E|C}\right)}^{t}\;V_{E|C}^{-1}\;\left({\underline{y}}_{E}-{\hat{\mu }}_{E|C}\right)/n_{E},$$

(A14) $${\hat{\mu }}_{E|C}=F_{E}{\underline{\hat{\beta }}}_{E}\;+\;V_{CE}^{t}\;V_{CC}^{-1}\;\left({\underline{y}}_{C}-F_{C}{\underline{\hat{\beta }}}_{C}\right).$$
